# Inflammation and Oxidative Stress in Obesity, Metabolic Syndrome, and Diabetes

**DOI:** 10.1155/2012/943706

**Published:** 2012-12-24

**Authors:** Pietro Galassetti

**Affiliations:** ICTS Metabolism/Bionutrition Core, University of California, Irvine, CA 92697-1385, USA

I do not think anybody even remotely connected with the field of diabetes, either as a researcher or as a health care provider, would have any serious objection to the concept that some degree of altered inflammatory activity or oxidative stress plays a serious role in multiple aspects of diabetes. Just to mention two of the best-known processes, a violent, acute inflammatory event leads to the destruction of beta cells at the onset of type 1 diabetes, and a chronic, subclinical proinflammatory state is at the base of the slow development of micro- and macrovascular complications accounting for the larger part of morbidity and mortality related to all forms of diabetes. This connection has led to a remarkable increase in research activity in this field in recent years. Running a PubMed search with “inflammation and diabetes” as search terms, for instance, returns 14,059 articles; “Oxidative stress and diabetes” 8850 articles; “inflammation and atherosclerosis” 11209 articles; and “inflammation and cardiovascular disease” 49245 articles. To a smaller extent, the very content of this special issue is a clear example of the variety and complexity of issues in the prevention, diagnosis, management, and therapy of diabetes, in which one or more inflammatory or oxidative stress component plays a critical role. 

 Inflammation and oxidative stress, however, are two extremely broad and comprehensive terms. This wealth of studies and results certainly clarified a large number of pathways, mediators and regulatory mechanisms, genes and posttranscriptional regulators of gene expression related to inflammation and oxidative stress. However, given the very large, and constantly growing, number of cell types and molecules involved, it often feels as though the rate at which new questions accumulate far exceeds the rate at which prior questions are definitively answered. We have now identified hundreds of cytokines and chemokines, whose full physiological function remains in many cases nebulous. Terms such as “pro” or “anti-inflammatory,” earlier closely associated with individual inflammatory mediators (such as the classically defined proinflammatory interleukin-6), are now used more reluctantly, as the same molecules often display less clear-cut activity, or even shift from one end of the spectrum to the other, depending on the surrounding metabolic or cell-signaling milieu. More importantly, often only some components of the complex inflammatory network are altered in a specific pathological condition. Identifying what these components are, and using them as biomarkers of onset, progression or response to treatment of a given condition has become one the main focuses of inflammatory research. Again, however, the immense diversity of these biomarkers renders the task extremely difficult. Let us hypothesize, for the sake of discussion, that a group of cytokines, or certain leukocyte surface markers of activation, are definitively demonstrated to increase significantly in a population of diabetic patients (let us say young caucasian adult males with type 2 diabetes), and that their increase is proportional to the degree of early endothelia dysfunction in these subjects. While this piece of knowledge can lead to therapeutic and preventive efforts in this specific populations, it immediately raises the issue of whether the same biomarkers are as effective in other populations, that is, in different age groups, gender, ethnicities, different degrees of diabetes control, different duration of the disease, and so forth. In short, each set of chosen inflammatory biomarkers should be closely tailored to as well defined a subpopulation of subjects as possible, which would obviously require an enormous amount of research work. This work is feasible and should indeed be planned and systematically performed. It is obvious, however, that to cover all aspects of the interaction between altered inflammation, oxidative stress, and diabetes (with associated prediabetic states such as obesity and metabolic syndrome), no single laboratory or group of laboratories can have the ability to perform all necessary studies. To really produce conclusive, reproducible, nonredundant results complementing each other and truly generating all interlocking pieces of an all-inclusive inflammatory jig-saw puzzle, a global scale, coordinated effort must be undertaken by a very large number of competent research groups, addressing in a synergistic, collaborative way the most pressing questions and then systematically chipping away at all collateral issues. Essential to this task is also the establishment of capillary informatics tools that would allow the immediate and complete exchange of data across groups, as soon as they are being gathered. While such an entity does not currently exist, at least not of the scale proposed here, many collaborations are indeed being formed across research groups, and the outlook for the future seems positive. In the meantime, I do not mean to discount the importance of many very good individual studies that are clarifying specific aspects of this broad issue. Given the immeasurable size of the task ahead, each new piece of information is precious, helpful, and welcome. 

*Pietro Galassetti* *Pietro Galassetti*

